# Height for age z score and cognitive function are associated with Academic performance among school children aged 8–11 years old

**DOI:** 10.1186/s13690-016-0129-9

**Published:** 2016-05-02

**Authors:** Demewoz Haile, Dabere Nigatu, Ketema Gashaw, Habtamu Demelash

**Affiliations:** School of Public Health, College of Medicine and Health Sciences, Bahir Dar University, Bahir Dar, Ethiopia; Department of Nursing College of Medicine and Health Sciences, Madawalabu University, Bale Goba, Ethiopia; Department of Public Health, College of Medicine and Health Sciences, Debre Tabor University, Debre Tabor, Ethiopia

## Abstract

**Background:**

Academic achievement of school age children can be affected by several factors such as nutritional status, demographics, and socioeconomic factors. Though evidence about the magnitude of malnutrition is well established in Ethiopia, there is a paucity of evidence about the association of nutritional status with academic performance among the nation’s school age children. Hence, this study aimed to determine how nutritional status and cognitive function are associated with academic performance of school children in Goba town, South East Ethiopia.

**Methods:**

An institution based cross-sectional study was conducted among 131 school age students from primary schools in Goba town enrolled during the 2013/2014 academic year. The nutritional status of students was assessed by anthropometric measurement, while the cognitive assessment was measured by the Kaufman Assessment Battery for Children (KABC-II) and Ravens colored progressive matrices (Raven’s CPM) tests. The academic performance of the school children was measured by collecting the preceding semester academic result from the school record. Descriptive statistics, bivariate and multivariable linear regression were used in the statistical analysis.

**Results:**

This study found a statistically significant positive association between all cognitive test scores and average academic performance except for number recall (*p* = 0.12) and hand movements (*p* = 0.08). The correlation between all cognitive test scores and mathematics score was found positive and statistically significant (*p* < 0.05). In the multivariable linear regression model, better wealth index was significantly associated with higher mathematics score (ß = 0.63; 95 % CI: 0.12–0.74). Similarly a unit change in height for age z score resulted in 2.11 unit change in mathematics score (ß = 2.11; 95 % CI: 0.002–4.21). A single unit change of wealth index resulted 0.53 unit changes in average score of all academic subjects among school age children (ß = 0.53; 95 % CI: 0.11–0.95). A single unit change of age resulted 3.23 unit change in average score of all academic subjects among school age children (ß = 3.23; 95 % CI: 1.20–5.27).

**Conclusion:**

Nutritional status (height for age Z score) and wealth could be modifiable factors to improve academic performance of school age children. Moreover, interventions to improve nutrition for mothers and children may be an important contributor to academic success and national economic growth in Ethiopia. Further study with strong design and large sample size is needed.

## Background

Academic achievement of school age children can be affected by several factors such as nutritional status, demographics, and socio-economic factors [1, 2]. No nation can afford to waste its greatest national resource: the intellectual power of its people. In many poor countries where malnutrition is widespread, it is considered a problem that negatively affects the ability of children to learn and causes them to perform at a lower level in school [3]. Studies have shown that food insecurity is associated with academic performance of school age children [4, 5].

An intervention study showed that free school breakfast programs resulted in significant improvements in attendance, mathematics grades, behavior and decreases in hunger [6]. Another study showed that improvement in academic performance would be achieved once proper nutrition is achieved [7]. Children who were malnourished in early life are more likely to have lower school attendance and to score poorly in cognitive tests during school age as compared to well-nourished children [8]. Many studies done in different parts of the world showed that poor growth characteristics are associated with poor academic performance. A prospective cohort study from Canada revealed that though overweight does not increase risk for poor educational outcomes, being underweight increase risk for poorer cognitive outcomes [9]. A study from Southeast Asia showed that all poor growth characteristics (HAZ, WAZ and BAZ) and non-verbal IQ scores were associated negatively [10]. A study from Southern Ethiopia also found that better HAZ and WAZ were positively correlated with cognitive performance tests [11].

Ethiopia is one of the sub-Saharan African countries largely affected by child malnutrition [12]. Studies conducted in different parts of Ethiopia have shown that under nutrition is common among school age children. A study from northwest Ethiopia revealed that 32.3 % of school age children were undernourished (27.1 % underweight and 11.2 % stunted) [13]. Another study from Gondar showed that the prevalence of stunting among school-aged children was 42.7 % in rural areas and 29.2 % in urban areas, while the corresponding figures for thinness were 21.6 and 20.8 % [14]. A study from eastern Ethiopia showed prevalence of stunting among school-aged children was 8.9 % of which 2 % had severe stunting [15]. A 2014 study in Addis Ababa, the nation’s capital found about 31 % of children were undernourished (19.6 % stunted, 15.9 % underweight) [16]. This implies that child malnutrition is still a public health problem in both urban and rural areas.

The future of a nation entirely depends on the welfare of its younger generation. Ethiopia is currently transitioning from the end of its first national growth and transformation plan to the beginning of its second growth and transformation plan, of which its success will largely depend on the academic performance of its school children and their future contributions. In 2012/2013 the net enrolment rate in Primary Level (1–8) was 85.9 %. Similarly, the net enrollment rate at Primary Level in the nation’s largest and most populous region, Oromiya region, was 83.9 %. The national repetition rate among primary school students was found to be 7.9 % while the repetition rate of Oromiya region was 9 %. The dropout rate for Oromiya was 18.8 % while the national was 15.7 % [17]. Therefore proper attention should be given to this group of children because their performance will impact the socioeconomic development of the entire country.

Though evidence about the magnitude of malnutrition is well established in Ethiopia, there is a paucity of evidence regarding how nutritional status is associated with academic performance among the nation’s school age children. Hence, this study is aimed to determine the association of nutritional status and cognitive performance with academic performance of school children in Goba town, South East Ethiopia.

## Methods

### Study area and period

An institutional based cross-sectional study design was employed in Goba town, Bale zone, Oromiya region, Southeast Ethiopia. The study was conducted from May to June 2014 among grade three elementary school students in Goba town. At the time the town had one preparatory school, two high schools, ten Kindergartens and 13 elementary schools, of which 7 were private and 6 were public [18]. The town schools also served the rural residents who live around the town. From these elementary schools, the study selected grade three students from randomly selected schools, in order to minimize inter-grade variability between different levels of grades. All of the student participants were of age 8 to 11 years old and had been enrolled in the school in 2013/2014 academic year.

### Sample size and sampling procedure

The sample size (n) was determined by using G power statistical software version 3.1.5 by assuming the effect size of 0.24, the level of significance (α), 0.05 (Zα_/2_ = 1.96) and the power (1 − *β* = 0.80) [19]. The study was conducted on 131 grade three school age children who were enrolled in elementary schools in Goba town. Initially the eligible schools were stratified into public schools and private schools by assuming socioeconomic difference between public and private school students. Two public and three private primary schools were then selected randomly. The total number and list of students were obtained from each selected school. To determine the number of students included in the study from each school, we used proportional allocation based on the number of eligible students from each school. For schools which have more than one section of grade three students, the same procedure was followed. Finally students were selected using a simple random sampling technique (computer generated random number). In any case when a randomly selected student was absent on three consecutive data collection days; he/she was substituted by the next randomly selected student from the same class room.

### Measurements

Socio-demographic characteristics were collected using structured questionnaire adopted from the Ethiopian demographic and health survey questionnaire [12]. Dietary intake was assessed using qualitative 24 h dietary recall method in a joint interview with both parent and child. From the qualitative 24 h dietary recall, dietary diversity for each student was calculated based on the WHO eight food groups [20]. The cognitive function of the students was assessed using selected tests from the Kaufman Assessment Battery for Children (KABC-II) and Raven’s Colored Progressive Matrices (RCPM) [21, 22]. The KABC II used Number Recall, Word Order and Hand Movement for measuring sequential processing (short term memory). In KABC II, simultaneous processing is measured by Rover and Triangles while planning (fluid reasoning) is assessed by using Pattern Reasoning. From KABC-II tests, core subsets for planning and conceptual thinking, were not administered because previous studies done in similar settings indicated that, most of the pictures in the subsets were not familiar to Ethiopian children [11, 23]. Tests for measuring learning and knowledge were not used for this study because they were not used previously in similar cultural settings. Raven’s Colored Progressive Matrices (RCPM) made up of three sets of twelve problems which measures the ability to solve problems and reasoning by analogy has been used extensively as a culturally fair test of intelligence [21]. Each of the cognitive tests (selected tests from KABC-II and RCPM) were administered by different well trained data collectors in separate class rooms with a quiet and distraction free environment. One data collector administered only one cognitive test for all study subjects to reduce inter individual differences. Anthropometric measurements (height and weight) were taken for all children included in the study. Body weight was recorded to the nearest 0.1 kg using the UNICEF SECA weighing scale. Instruments were checked daily against a standard weight for accuracy. Calibration of the indicator against zero reading was checked before weighing every child. Children were weighed with light clothing and without shoes. Height was measured to the nearest 0.1 cm using the Shorr measuring board without shoes at Frankfort plane standing position. The age of children in completed years was obtained from school and confirmed from their parents. The academic performance of the school children was measured by collecting the preceding semester academic result from the school record. We used the students’ mathematics and overall average semester results to measure academic performance.

### Data entry, processing and analysis

Data were checked for completeness, cleaned, coded and entered into SPSS version 20 and WHO AnthroPlus Version 1.0.4 for analysis. The nutritional status of height-for-age, weight-for-age, and weight-for-height were calculated from measurements using WHO AnthroPlus and compared with reference data according to the WHO 2006 population. Children below negative 2 standard deviation (−2SD) according to the WHO median for weight-for-age, height-for-age and weight-for-height were considered under-weight, stunted or wasted, respectively. Normal was defined as Z-score greater than or equal to -2SD. A descriptive analysis was conducted to obtain summary statistics (frequencies, means and standard deviations). Pearson correlation was used to check the relationship between nutritional status and academic performance. The association between academic performance and cognitive performance was also measured by Pearson correlation. Those variables which were found statistically significant at P value less than 0.25 in the bivariate analysis were entered into the multivariable linear regression model to identify the independent predictors of academic performance. This cutoff point prevented removing variables that would potentially have an effect during multivariable analysis [24]. Variables with p-value less than 0.05 in the final multivariable model were accepted as statistically significant and declared as associated factors.

### Ethical consideration

The ethical clearance was obtained from the institutional review board of Madawalabu University. A letter of permission was also obtained from the Goba town education office. Informed written consent was obtained from all parents (mothers/caregivers) after explanation of the study objectives, study period and measurement procedures. The willingness of the school age children was also asked in addition to parents consent. The students and parents were assured that the information they provided would be kept confidential.

## Results

### Socio-demographic characteristics

The majority of the study participants were Orthodox (97,74.8 %) by religion and Oromo by ethic group (66, 51.1 %). Ninety five (74.8 %) and 122 (93.1 %) of the school age children were from families who were currently on marriage and residing in urban areas, respectively. Nearly one third of (42,32.8 %) the children were from households headed by husbands. Fifty four (47.0 %) of the school age children had a father who attended formal education up to the level of high school. Nearly two thirds (84, 64.9 %) of the school age children were living with both their fathers and mothers. Nineteen (14.5 %) of school age children were living with their grandparents. Eighty four (63.4 %) of the school age children were male by sex. The mean (±SD) age of the school age children was 10.02 (±0.86) years (Table 1).

**Table 1:** Socio-demographic and economic characteristics of respondents in Goba Town, South east Ethiopia, 2014

Variables		Frequency	Percent
Religion	Orthodox	97	74.8
	Muslim	27	20.6
	Protestant	6	4.6
Ethnic group	Oromo	66	51.1
	Amhara	60	45.8
	Others^b^	4	3.1
Marital status	Married	95	74.8
	Divorced	6	4.6
	Separated	14	10.7
	Widowed	13	9.9
Head of the household	Husband	42	32.8
	Wife	17	13.0
	Both	54	41.2
	Others^c^	17	13
Place of residence	Urban	122	93.1
	Rural	9	6.9
Educational status of the father	Illiterate	12	10.3
	Primary (1–8)	24	20.5
	Secondary school (9–12)	54	47.0
	Tertiary (>12)	26	22.2
Educational status of the mother	Illiterate	13	9.9
	Primary (1–8)	43	32.8
	Secondary (9–12)	51	38.9
	Tertiary (>12)	17	13.0
Wealth index	Poor	46	36.8
	Medium	35	28.0
	High	44	35.2
With whom the child lives	Father only	4	3.1
	Mother only	19	14.5
	With both	84	64.9
	Grand parents	19	14.5
	Others^a^	4	3.1
Sex	Male	83	63.4
	Female	48	36.6
School type	Private schools	67	51.1
	Governmental schools	64	48.9
Family size	≤3	32	24.6
	4–5	61	46.9
	≥6	37	28.5

^a^Aunts, sister, religious brothers, any non relatives ^b^Gurage, Tigre Wolayita ^c^grandparents, relatives, aunts

There was a statistically significant positive association between all cognitive test scores and average academic performance except for number recall (*p* = 0.12) and hand movements (*p* = 0.08). This study found that there was a statistically significant positive correlation between all cognitive test scores and mathematics score (*p* < 0.05) (Table 2).

**Table 2:** Correlation between cognitive function test and academic performance among school aged children in Goba Town, South east Ethiopia, May 2014

Cognitive test scores		Academic performance	
		Average semester result	Mathematics
Number Recall score	r	0.14	0.19*
	*p*-value	0.12	0.03
	N	131	130
Rovers score	r	0.22*	0.22*
	*P*-value	.013	0.01
	N	131	130
Hand Movement score	r	0.16	0.20*
	*P*-value	0.08	0.03
	N	131	130
Pattern score	r	0.24**	0.27**
	*P*-value	0.005	0.002
	N	131	130
Word Order score	r	0.23**	0.19*
	*p*-value	0.008	0.028
	N	131	130
Triangles test score	r	0.33**	0.29**
	*p*-value	0.001	0.001
	N	131	130
Ravens CPM test score	r	0.38**	0.38**
	*p*-value	0.001	<0.001
	N	129	128

*Statistically significant at p <0.05, **Statistically significant at p<0.01

As showed in Table 3, there was a statistically significant positive correlation between height for age Z score (HAZ) and mathematics score among school aged children (*p* = 0.026). However, both weight for age Z score (WAZ),and body mass index for age Z score (BAZ) had no statistically significant association with academic performance (average semester result and mathematics score) (*p* > 0.05).

**Table 3:** Correlation between anthropometric Z scores and academic Achievement among school aged children in Goba Town, South east Ethiopia, May 2014

Anthropometric z scores		Academic performance	
		Average semester score	Mathematics score
HAZ^a^	r	0.103	0.197^a^
	*p*-value	0.249	0.026
	N	128	127
WAZ^b^	r	0.061	0.099
	*P*-value	0.497	0.270
	N	127	126
BAZ^c^	r	−0.029	−0.061
	*p*-value	0.743	0.496
	N	127	126

^a^Height for Age Z score, ^b^Weight for Age Z score, ^c^Body mass for age Z score

Variables including residence, maternal education, paternal education, diet diversity, meal frequency, breakfast habit, iodized salt consumption, sex of the child, occupation, attendance of preschool program and family size were not significantly associated with academic performance (*P* > 0.25). Hence those variables were excluded from the multivariable linear regression model.

In the bivariate analysis only height for age Z score and wealth index were significantly associated with mathematics score, qualifying as candidate variables for the multivariable linear regression model. Age and wealth index were positively associated with average score of all academic subjects (courses) of the preceding semester in the multivariable model.

In the multivariable linear regression model better wealth index was significantly associated with better mathematics score (ß = 0.63; 95 % CI: 0.12–0.74). A single unit change of wealth index resulted in 0.63 unit change in mathematics score. Height for age Z score was found positively associated with mathematics score in the multivariable model. A single unit change in height for age Z score resulted in 2.11 unit change in mathematics score (ß = 2.11; 95 % CI: 0.002–4.21). A single unit change in wealth index resulted in 0.53 unit change in average score of all academic subjects among school age children (ß = 0.53; 95 % CI: 0.11–0.95). Older children scored significantly higher in average semester result. A single unit change of age results 3.23 unit change in average score of all academic subjects among school age children (ß = 3.23; 95 % CI: 1.20–5.27).

## Discussion

This study found that WAZ and BAZ were not significantly associated with average semester results or mathematics score among school age children, age 8–11 years. Similarly a study from Malaysia found no association between poor nutritional status and school performance among a sample of 7 to 8 years old primary school children [25]. However, in contrast to the finding of this study, studies have shown that higher WAZ is positively associated with academic performance [26, 27]. High WAZ score is associated with better cognitive performance [11]. The small sample size of this study possibly a reason for absence of association between WAZ and academic performance. Additionally, this study was conducted among school age children attending urban schools, where prevalence of underweight is tends to be lower than in rural schools. Therefore the effect of underweight on academic performance in a study population where malnutrition prevalence is low might not reflect the effect at a population level. The absence of association between BAZ and academic performance might be explained by the fact that BAZ indicates acute nutritional status and do not interfere with cognitive functioning.

This study found that good HAZ score was significantly associated with higher mathematics scores. Similar finding came from a study conducted in Sri Lanka which showed that higher HAZ score is associated with better academic score [27]. Height-for-age reflects the accumulation of nutritional deprivation throughout the years, which may consequently affect educational achievement of children [28]. A study from Uganda revealed that HAZ had statistically significant positive associations with learning achievement in English (language) and mathematics among grade 4 children [29]. A study among grade 4 children in Sri Lanka showed a significant positive association between height-for-age and examination scores [30]. Stunted children have shown more anxiety, depression and lower self-esteem than their non stunted counter parts [31]. Similarly, protein malnutrition (and low energy availability in general) may have negative effects on brain development. Chronic protein energy malnutrition (stunting) affects the ongoing development of higher cognitive processes during childhood years [32, 33]. Stunted children have impaired behavioral development in early life [34] and have poorer cognitive ability [35] than non-stunted children. Brain functions such as cognition, memory and locomotors skills are affected by under nutrition [36, 37]. However, the associations between poor linear growth and impaired neurodevelopment are not well understood [36]. Cognitive functioning might mediate the association between stunting and academic performance. This study found that all the cognitive test scores were significantly associated with the mathematics scores. Except number recall and hand movement, all other cognitive tests were correlated with average semester results.

This study found that higher wealth index is associated with better mathematics score of school age children. Many consistent findings were reported from different part of the world. Food security, which is correlated with wealth index has predicted impaired academic performance in mathematics for girls and boys [4]. A meta analysis study showed that there is a strong correlation between socio economic status and academic performance [38]. Poor socioeconomic condition of the family is one of the determinants for poor school performance among children [39]. Primary school children in Malaysia from the lower income group had significantly poorer academic performance [2]. The association between socio economic status and academic achievement widens with increasing age. Socio‐economic gaps in the early school years has long lasting consequences. Particularly, as children in low socio economic status get older, their situation tends to worsen [40]. The association between socioeconomic status and academic achievement is complex and included several factors such as nutrition, school environment, home living environment, material support etc. Socio economic status might affect educational performance due to its influence on affordability of quality of residence, access and affordability of Information Communication facilities and services, and library materials which deal with academic matters [41]. Low socioeconomic status is associated with chronic stress which has long term negative consequences on brain development [42]. Furthermore, low socioeconomic individuals have high levels of stress hormones such as cortisol and catecholamines [43].

This study implies that academic performance is affected by chronic malnutrition and wealth. The education sector, which largely determines the welfare of a country, can be improved by investing in nutrition, particularly on chronic malnutrition. Stunting begins in utero and continues for at least the first 2 years of postnatal life; the period from conception to a child’s second birthday (the first one thousand days) has therefore been identified as the most critical window of opportunity for interventions. There are proven interventions to prevent stunting such as exclusive breastfeeding, complementary feeding, infection prevention, sanitation and hygiene. Nutrition interventions should also be strengthened in schools to improve children’s academic performance. Improving the socioeconomic status of a community has profitable gains in terms of students’ academic performance and development of the nation.

This study had some limitations. The first limitation was its small sample size and biased inclusion of children mostly from urban residency. This study used only anthropomorphic measurements and did not assess the micronutrients status of study participants. Furthermore, there may have been differences in the evaluation system for students’ academic performance among the public and private schools. Finally, physical performance capacity and motor skills were not measured in this study.

## Conclusion

Nutritional status and wealth could be additional modifiable factors to improve academic performance of the children. Moreover, interventions to improve nutrition for mothers and children may be an important contributor to academic success and national economic growth in Ethiopia Further study with a strong design and large sample size is important.
